# William Budd

**Published:** 1967-07

**Authors:** R. C. Wofinden


### WILLIAM BUDD

R. C. WOFINDEN

"The idea that phthisis is a self-propagated zymotic disease, and that all the leading phenomena of its distribution may be explained by supposing that it is disseminated through society by specific germs contained in the tuberculous matter cast off by persons already suffering from the disease, first came to my mind, unbidden, so to speak, while I was walking on the Observatory Hill at Clifton, in the second week of August 1856".

This announcement was made by Dr. William Budd in a memorandum which was published in the Lancet in 1867. Although the infectious nature of phthisis had been recognised three to four hundred years B.C., it had been largely forgotten. Indeed, Budd's paper in which he outlined his reasons for the communicability of phthisis, was received with scorn by most of his colleagues in this country and hailed by only a few as an original idea.

Who was this man, who, contrary to the current miasmatic theory of the origin of infections, talked and wrote in terms of germs and the specificity of such diseases? In his prime, his contemporaries described him as "of a good height, well made and of a brave presence with a strong looking, robust body" and as "vivacious, eloquent, imaginative and genial".

He was born in Devon in 1811 and was one of a family of ten children whose father, Samuel, was a country surgeon in North Tawton. His mother, Catherine, was reputed to be "a little black, wiry Dartmoor woman" . . . who took a very active part in the direction of the affairs of her family". Whether hers was the guiding hand or not, she and Samuel produced a remarkable family and no fewer than six of their sons entered the medical profession. Four of the brothers practised in Devonshire, the despair of competing doctors who were convinced that all the potential patients were confirmed Buddists!

William studied medicine over a period of 10 years in London, Paris, and Edinburgh where he finally graduated as a doctor of medicine in 1838 at the age of 29. It is almost certain that his studies in Paris were interrupted at least once, by an attack of typhoid fever for which he was treated by copious blood letting by one of his teachers, Broussais. He came to Bristol in 1841 and put up his plate at 22 Park Street now occupied by George's Bookshop, and later moved to Clifton, first to 13 Lansdown Place and then to The Manor House which is now a University hostel for students.

He must have made a name for himself fairly quickly, for in the following year he was appointed physician to St. Peter's Hospital—a post regarded as a "stepping stone towards the coveted appointment of physician to the Infirmary". This hospital stood beside St. Peter's Church, unhappily destroyed in the blitz. It was built probably in the 15th century as a private house and rebuilt in 1612. In the reign of William III it was used as a Royal Mint but in 1697 it was converted to a hospital for the sick poor. Its origin sprang from the formation, in 1696, of the "Bristol Incorporation of the Poor", a body formed to deal with the problem of poor relief. The main aims of the Incorporation were to provide hospital treatment for the sick poor, the creation of a Union common fund for relief purposes, the combination of employment and industrial training with charity and the suppression of mendicancy by drastic punishment. One of its earliest physicians was the famous Dr. Thomas Dover and it was undoubtedly used for educating medical students. In Budd's day there were reputed to be 200 beds for medical and surgical cases. However, some years afterwards, when he had moved on to the Infirmary, it is alleged that 600 men, women and children were herded together in the building, and that during one of the Bristol cholera outbreaks there were 168 victims. Until 1861 lunatics and fever cases were treated there and the overcrowding was not relieved until the French prison at Stapleton had been adapted for hospital purposes.

There can be little doubt that at St. Peter's Hospital Budd had ample opportunity for studying at first hand most, if not all, of the epidemic infections of that period—typhoid and typhus fever, smallpox, cholera and the like. But, as we shall see, he was not a physician tied to his hospital beds and showing no interest in the lives and leisure of his fellow citizens.

Let us pause for a moment and consider the contemporary scene in which he worked. It will be recalled that the year he was appointed as physician to St. Peter's was also the year in which Chadwick published his famous "Report on the Sanitary Condition of the Labouring Population of Great Britain". This report undoubtedly led to the setting up of a Health of Towns Commission in the following year and Bristol was one of the towns to be investigated.

Here was a city for many centuries second only to London in commerce, population and wealth. From surrounding eminences one could see a city astride a river and adorned with the towers and graceful spires of its 36 churches. Its main suburb of Clifton was celebrated as a place of health and recreation. But there was a darker side to the picture, particularly in the old City with its narrow, unpaved, unlit, and tortuous streets shut in by ancient houses—much admired by those who did not live in them. Water was a scarce and costly commodity; the Frome and Avon rivers were, as they still are, the *cloaca maxima* for sewage disposal and the burial grounds were overful with rotting corpses. In lower Clifton (Hotwells) each small house was occupied by two to three numerous families. Animal food rarely entered into their diet and their persons, "except on holidays and Sundays were at all times squalid, filthy from the neglect of ablution and sordid from the miserable garments they were covered with". Small wonder that at that time Bristol's death rate of 31 per 1,000 was exceeded by only two other big towns, Liverpool with a rate of 35 per 1,000 and Manchester with a rate of 32 per 1,000.

Budd was one of several eminent Bristol medical men who gave evidence before the Health of Towns Commission. He was an excellent witness, producing many graphic descriptions of the appalling conditions under which many of the citizens lived—conditions which made them "filthy, coarse and brutalised"—and drove them to the beer houses for they could not stand the abominable stench within their own homes. Here is a description which he gave about the houses abutting the River Frome:—

> "At the back of each is a gallery, built of wood which, projects from the wall of the house and overhangs the ditch. In the corner of each gallery is a privy. From both there is a view of a range of other privies up and down the stream belonging to the houses which abut upon it. These privies hang over a bank of mud, the level of which is only swept at spring tide, or when the Frome is swollen with freshets. The state of things in the interval is too loathsome and disgusting to describe ".

He reported that on one occasion he found four cases of contagious fever occupying one bed and it was not uncommon to keep corpses for 12 days in the same room as the family ate and slept in, because they could not afford to take time off for the funeral.

Water supplies in Bristol in those days were hopelessly inadequate. William Kay gave evidence in 1845 that only 5,000 persons, constituting the wealthy families of Bristol and Clifton had piped water supplies in their houses, while over 73,000 had to rely on Waterleders (who transported the filthy water of the Avon or Frome at 2d. per family per day) or on public or private wells. Many of the wells, particularly in the low-lying areas, were polluted by cess-pool fluids. Indeed the commissioners were told that " The water from St. Peter's well is considered better for making tea than those near it, a characteristic perhaps due to the percolation to it of the filthy waters of the Moat. When the Moat water has been out, this well has been known to be pumped dry ". Most of the wells and springs have now disappeared, but a few, such as St. John's Conduit in Nelson Street, still survive. This conduit, used as a source of supply in the last war, taps springs on the north-east side of Brandon Hill and the top of Park Street; originally they fed the Carmelite Friary which used to stand where the South Western Gas Board stood; and later a " feather pipe " extended the supply to St. John's.

At this time, Chadwick, Simon and others believed that fevers were of miasmatic origin—the results of the poisonous exhalations from putrifying organic matter. Typhoid and typhus fevers were considered to be but two among many forms of " the fever " which arose by spontaneous generation. Even before he came to Bristol, Budd was convinced that this was not so. He taught his students that these two diseases could be distinguished, clinically, epidemiologically, and at post-mortem. He was sure, from his experience before he came to Bristol that typhus could only be spread by contagion whereas typhoid was often caused by drinking water contaminated by the intestinal discharge from patients. Further years of practical experience in Bristol confirmed him in this view in the Richmond Terrace and Kingswood outbreaks.

Budd was amongst the few of his day who believed in the animate nature of infection. In 1849 cholera broke out in Bristol and he seized the opportunity of first-hand study. In that year he published his first paper on infectious disease—" Malignant cholera—its mode of propagation and its Prevention ". He was convinced that cholera was due to a living " organism " of distinct species. Budd and his colleagues Brittan and Swayne claimed to have seen under the microscope " fungi " which they had isolated from the stools of a cholera patient and which they thought was the causal agent which could be taken into the body by drinking infected water. Budd delayed publishing his paper in order to let Dr. Brittan announce his observation. It was about the same time that Dr. John Snow had been pursuing his inquiries into the London cholera outbreak and his paper, embodying his brilliant researches, was published just before Budd's paper appeared. Both can therefore be considered to have arrived independently at the same con-

* Now the Transport and Cleansing Department.

clusion—that infected water supplies can cause cholera; but history awards the credit to Snow. Unfortunately too, for Budd, the cholera " fungi " were soon shown to consist of epithelial cells, starch granules and fat globules.

However, Budd must be given the credit for stressing the need to disinfect the stools of cholera patients to kill the infection at its source. He was a strong advocate also of disinfection of the drains and sewers during epidemics and of the patients' clothing and bedding and of the corpse; he also advised the boiling of milk and water which might have been contaminated. Indeed, so strongly did he believe in disinfection that he persuaded the City Guardians to set up depots of chemicals and disinfectants which citizens were exhorted to draw upon and use, free of charge. In the 1866 cholera epidemic, Budd in association with the recently appointed Medical Officer of Health, Mr. David Davies, and Mrs. Norris an " intelligent woman ", was appointed to visit the poor each day to see that disinfection was carried out. This was so successful that money was raised by special subscription and very soon 28 women, each with an allotted district, were employed on this work. This practice had no support in Great Britain, but Budd claimed that by its implementation in the 1854 and 1866 epidemics the mortality rate from cholera in Bristol was reduced to one fourteenth of that experienced by other towns. To use his words " In Bristol the seed has been destroyed and the crop has failed ".

In view of his convictions about the role of polluted water supplies in the dissemination of typhoid and cholera it is not surprising to find that he took an active part in 1844 in the formation of the Bristol Waterworks Company. Their first meeting was held in the White Lion Hotel, now the Grand Hotel—the scene of many a convivial evening for the B.R.I. consultants. His participation was contingent upon the company following his two principles—that their water must be drawn from sources beyond the reach of sewage contamination and that it should be distributed under the constant pressure system. The company never had cause to regret following his policy for at no time since their formation has their supply caused any waterborne infections in Bristol.

Let it not be thought, however, that Budd was interested only in intestinal infections. Some of his observations on phthisis have already been quoted and he also wrote papers on scarlet fever, and anthrax, as well as on animal infections. In his memorandum on phthisis Budd outlined the evidence which led him to suppose it to be a true zymotic disease :—

(a) Actual instances in which there was evidence to show that phthisis was communicated from one person to another.

(b) Its greater prevalence in crowded communities—convents, barracks, prisons, etc.—which were known to favour the spread of zymotic diseases generally.

(c) Its geographical distribution in past and present times. Here he instanced the devastating effect of tubercle on " unsalted " populations—in the South Sea Islands and the African seaboard. To use his words :—

> "The contrast between original entire immunity and present extreme fatality is very striking, and can only be rationally explained by the importation of a new and specific morbid germ " . . . " Try every other supposition and the facts are inexplicable; make this one supposition, and they are at once explained ".

But perhaps his most important claim to fame was his enunciation of the underlying principles of infectious diseases—their specificity, the nature of the agent of infection and the absurdity of the current doctrine of spontaneous generation, the channels by which infection was spread and schemes for the investigation of epidemic and epizootic diseases. His paper on variola ovina (sheep small pox) was considered by Goodall to have "anticipated in the main principle the modern experimental epidemiologists with their herds of mice". Reading this paper today, of his coach journey to the remote Wiltshire farm (perhaps in the same coach as the one now modelled in the City Museum) in order to study the out-break on the spot, one cannot help feeling the present-day need for a few more "shoe-leather" instead of "arm-chair" epidemiologists.

Few honours were bestowed upon him; in 1871 he was made an F.R.S. some two years before he was laid aside with a right-sided hemiplegia. For seven years he lingered on. Death took him in 1880 when he was in his 69th year. He died at Clevedon and was buried in Arno's Vale Cemetery in Bristol.

Major Greenwood described Budd as "almost the last of the old race of epidemiologists" but Goodall, from whose book I have freely borrowed, judged him, and in my opinion rightly so, to be the first of the modern epidemiologists. As Goodall says :—

> "When William Budd came on the scene epidemiologists wandered darkling, 'in a gloomy wood astray', a wood obscured by hazy miasma and nebulous constitutions, in which all sorts of seductive phantasms were ever looming up, seemingly with effortless spontaneity, to mislead the wanderer. This darkness he lightened with the penetrating rays of rational deduction from observation, and showed the way to the open fields where views were wider and the vision clearer".

It is sad to reflect that from such a brilliant family there are no survivors bearing the name of Budd. We in Bristol are proud of his achievements. It is fitting that his name has been perpetuated in the name of a ward in the Royal Infirmary and in the William Budd Health Centre, the first to be erected under the National Health Service Act; even more fitting that one of his grandsons, the late Mr. A. J. Wright, should have been the Chairman of the Health Committee at the time the health centre was planned and built.

